# On the Interplay of DLIP and LIPSS Upon Ultra-Short Laser Pulse Irradiation

**DOI:** 10.3390/ma12071018

**Published:** 2019-03-27

**Authors:** Sabri Alamri, Fotis Fraggelakis, Tim Kunze, Benjamin Krupop, Girolamo Mincuzzi, Rainer Kling, Andrés Fabián Lasagni

**Affiliations:** 1 Fraunhofer Institute for Material and Beam Technology IWS, Winterbergstrasse 28, 01277 Dresden, Germany; tim.kunze@iws.fraunhofer.de (T.K.); benjamin.krupop@iws.fraunhofer.de (B.K.); andres_fabian.lasagni@tu-dresden.de (A.F.L.); 2 ALPhANOV, Technological Centre for Optics and Lasers, Optic Institute of Aquitaine, rue F. Mitterrand, 33400 Talence, France; Girolamo.mincuzzi@alphanov.com (G.M.); Rainer.kling@alphanov.com (R.K.); 3 CELIA, University of Bordeaux-CNRS-CEA UMR5107, 33405 Talence, France; 4 Institute for Manufacturing Technology, Technische Universität Dresden, George-Baehr-Str. 3c, 01069 Dresden, Germany

**Keywords:** direct laser interference patterning, LIPSS, USP laser source, stainless steel, polyimide, sapphire

## Abstract

Controlling laser induced surface morphology is essential for developing specialized functional surfaces. This work presents novel, multi-scale periodic patterns with two-dimensional symmetry generated on stainless steel, polyimide and sapphire. The microstructures were realized by combining Direct Laser Interference Patterning with the generation of Laser Induced Periodic Surface Structures in a one-step process. An industrial, fiber femtosecond laser source emitting at 1030 nm with a pulse duration of 500 fs was utilized for the experiments. In the case of stainless steel, it was possible to create line-like or pillar-like surface patterns by rotating the polarization orientation with respect to the interference pattern. In the case of polyimide and sapphire, the absorption of the laser radiation was promoted by a multiphoton mechanism. In polyimide, grooves and pillars of several microns in depth were produced over an area much larger than the spot size. Finally, for sapphire, the simultaneous generation of interference-like pattern and laser induced periodic surface structures was realized. The results reported here provide valuable data on the feasibility to combine two state-of-the-art techniques with an industrial apparatus, to control the induced surface morphology.

## 1. Introduction

In the last decades, several aspects have increased the interest in ultrashort-pulsed laser (USP) material processing [1]. Across industries, feature sizes are becoming smaller, which demands advanced USP laser processes for precise material machining and manipulation [2]. Several applications employing USP lasers have been established in the area of surface functionalization. For instance, precise laser machining methods have been utilized to produce structural colors [3,4], to control the surface wettability [5], to achieve directional water transport [6] as well as to tune the adhesion of cells and bacteria for biomedical applications [7,8], and to control surface friction and wear properties [9,10,11,12,13]. Furthermore, several examples in nature have shown that even better performances can be obtained combining surface features with different length-scales, e.g., by producing nanostructures on top of micro features (hierarchical surface patterns) [14].

To produce surface textures with feature-sizes in the sub-micrometer range, different approaches have been reported. For example, laser induced periodic surface structures (LIPSS) occur on solids upon irradiation with polarized lasers [15]. LIPSS usually emerge as a relief composed of (quasi-)periodic features, which exhibits a clear correlation with the wavelength λ and polarization of the radiation [16,17]. These structures can be generated on almost any material (metals, semiconductors, and dielectrics) [18] and for a huge range of pulse durations [19]. 2D-LIPSS have been also reported after surface irradiation by crossed and circularly polarized double pulses, upon variation of the intra-pulse delay in picosecond and nanosecond regime [20].

Depending on the material and the irradiation conditions (e.g., laser fluence (Φ), number of laser pulses (N), pulse separation, pulse duration, laser wavelength and polarization) different types of LIPSS will emerge. For example, low spatial frequency LIPSS (LSFL) have been observed on strongly absorbing materials (semiconductors and metals), which are perpendicularly oriented to the laser’s polarization [21]. In addition to LSFL, high spatial frequency LIPSS (HSFL) with periods smaller than half of the wavelength have been fabricated at fluence values that are very close to the material damage threshold, for pulse durations in the fs- to ps range [18]. The HSFL have either a parallel or perpendicular orientation to the laser beam polarization. Another approach used to produce deterministic periodic textures with feature size down to the sub-micrometer range is direct laser interference patterning (DLIP) [22,23]. It consists in creating interference patterns by overlapping two or more laser beams and with this directly treating the material’s surface [23].

In this study, we investigate the super-positioning of DLIP and LIPSS methods to generate repetitive surface structures on stainless steel, polyimide and sapphire. The morphology of the produced surface textures was investigated by white light interferometry and scanning electron microscopy. Fourier transform analyses were utilized for measuring the period of the structures.

## 2. Materials and Methods

**Figure 1:**
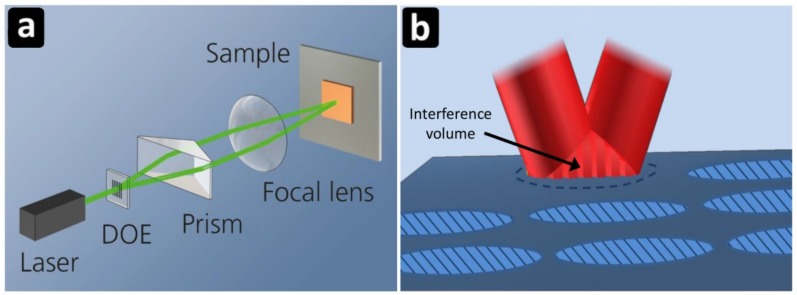
Scheme of the employed experimental setup (**a**) and descriptive explanation of the structuring method (**b**).

The laser processing experiments were performed on commercial 316 stainless steel plates, sapphire substrates as well as on polyimide foils. The stainless steel samples had dimensions of 20 × 20 × 0.5 mm^3^. The sapphire wafers had a diameter of 50.8 mm and 0.4 mm thickness. No cleaning process took place before irradiation. The structuring of the samples was carried out in ambient air conditions. The interference experiments were conducted using a compact DLIP module (developed by Fraunhofer IWS, Figure 1a) designed to produce line-like periodic patterns with spatial periods between 1.0 and 10 µm. The DLIP optical module was combined with an industrial fs laser (Tangor HP, Amplitude Systemes, Pessac, France) operating at a wavelength of 1030 nm, providing 500 fs pulses with pulse energies up to 300 µJ and a TEM00 beam shape. The resulting beam size at the interference position (section of the interference volume, Figure 1b) has been measured with a beam camera. A three-axis stage (ALIO AI-LM-20000-XY-I ULTRA) with linear repeatability of 30 nm was employed for positioning the sample, and the DLIP module during the structuring processes, displacing the DLIP-pixels with a defined distance.

The morphology of structured samples was characterized using white light interferometry (Leica DCM 3D, Leica Camera, Wetzlar, Germany) employing a 50× magnification objective, with lateral and vertical resolution of 340 nm and 4 nm, respectively. Topographical measurements have been carried out using a scanning electron microscope (SEM) provided by JEOL (JSM 6610LV, Tokyo, Japan). The period of the structures was defined by Fourier Transform (FT) analysis of the SEM images utilizing the open source software Gwyddion 2.50.

The parameters used for texturing, namely the laser fluence (Φ), the DLIP period (λ_DLIP_) and the incident number of pulses (N) are summarized in Table 1.

## 3. Results and Discussion

### 3.1. Stainless Steel

**Figure 2:**
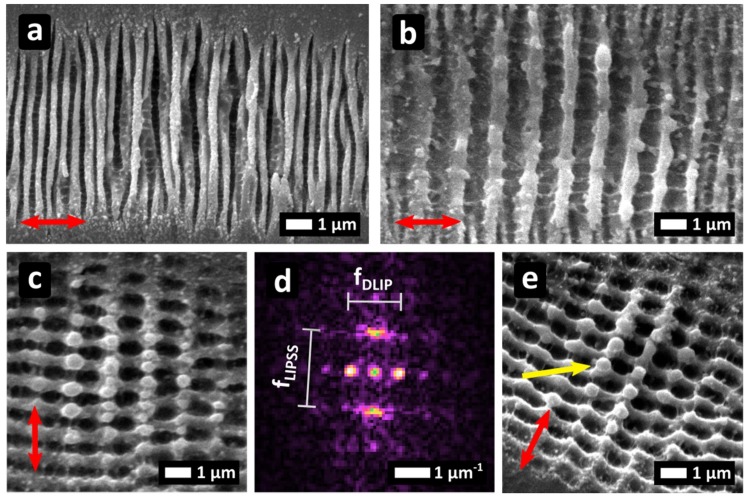
Stainless steel substrates irradiated with: (**a**) Φ = 0.2 J/cm^2^ and N = 50; (**b**) Φ = 0.5 J/cm^2^ and N = 20; (**c**,**e**) Φ = 0.4 J/cm^2^, N = 20; (**d**) Fourier Transform of (c); In (**e**), the sample was tilted. The red arrow indicates the beam polarization.

Stainless steel substrates were irradiated using the two-beam DLIP configuration and SEM images of the surface processed under different conditions are shown in Figure 2. The chosen spatial period for DLIP was selected to be comparable with the employed laser wavelength. Two polarization orientations were considered; parallel (I_∥_P) and perpendicular (I_⊥_P) with respect to the direction of the interference pattern. After finding a parameters window in which the inference patterning and the LIPSS formation occurs, a first set of experiments was carried out to investigate the effects of the laser fluence Φ and the number of laser pulses (N). A linear polarization perpendicular to the interference pattern has been selected and two structuring conditions have been employed, producing the same amount of cumulative fluence dose D = 10 J/cm².

When low laser fluences and a high number of pulses are used (Figure 2a, Φ = 0.2 J/cm^2^ and N = 50), the LIPSS formation is dominant and shallow DLIP microstructures are produced. The produced LIPSS are the predominant morphology especially in the center of the crater with period of Λ_LSFL_ = 480 ± 7 nm formed perpendicular to the polarization orientation, which is about the half of the period that can be expected by LSFL generation only. Simultaneously a period of Λ_DLIP_ = 1.51 ± 0.04 μm corresponding to the DLIP structures was produced. In the outer rim of the crater solely LIPSS were formed, with smaller period (Λ_LSFL_ = 440 ± 2 nm) than typical LSFL on stainless steel, which often is in the 900 nm rage [18].

When the fluence level is increased by a factor of 2.5 (Φ = 0.5 J/cm^2^) and the number of pulses are reduced to N = 20 delivering the same total dose (Figure 2b), predominant DLIP structures were formed in the center of the crater. Their period was Λ_DLIP_ = 1.308 ± 0.006 μm, smaller compared to Figure 2a, with δΛ_DLIP_ = 13%. In the outer rim of the crater in Figure 2b, LIPSS were formed with a period of Λ_LSFL_ = 444 ± 8 nm.

Conclusively, for the same dose, low Φ and large N values, favour the LSFL formation (Figure 2a) whilst higher Φ and smaller N values, favour the DLIP interaction (Figure 2b). The Λ_LSFL_ variation between the center and the outer part of the crater as well as the Λ_DLIP_ variation between Figure 2a and 2b is attributed to the interplay between the two superimposed interaction mechanisms forming textures in the same direction. Though DLIP and LIPSS formation are in competition, their formation mechanisms are similar and can be related to the inhomogeneous light absorption, which triggers a microfluidic movement of the molten material. Further theoretical and experimental investigations are necessary in order to understand the mechanisms which determine the period of the induced microstructures in this configuration.

Figure 2c, illustrates the I_⊥_P case for Φ = 0.4 J/cm^2^, N = 20 and D = 8 J/cm^2^. In contrast with the former configuration (I_∥_P), here, DLIP and LSFL formation mechanisms are perpendicular to each other and features corresponding to both are simultaneously visible. A 2D pattern is formed on the surface (Figure 2c–e) pointing out that the DLIP and LSFL formation is not in competition as for I_∥_P. The 2D-FT map of Figure 2c is shown in Figure 2d, indicating the spatial frequencies of the structures. For the LSFL, Λ_LSFL_ = 936 ± 19 nm and for the DLIP, Λ_DLIP_ = 1442 ± 16 nm was derived from the Fourier analysis, which perfectly matches the expected periods of each individual process. In the outer rim of the crater, LSFL period was Λ_LSFL_ = 851 ± 1 nm smaller than in the center. Interestingly, upon tilted sample observation (Figure 2e), nanoscale round protrusions (yellow arrow) were observed for the first time to our knowledge.

The formation of LIPSS and DLIP for I_⊥_P could be explained with a mechanism similar to what was previously proposed. For the LIPSS formation the mechanism includes two steps. In the first one, during laser irradiation, the light is inhomogeneously absorbed due to surface plasmon excitation [24]. In a second step, the inhomogeneously heated surface is subjected to microfluidic movement driven by temperature gradients leading to LIPSS formation [25,26]. Nevertheless, since a larger periodic intensity modulation is superimposed (DLIP pattern), the microfluidic material flow is driven by the induced surface temperature gradients between the interference maxima. The coexistence of DLIP and LIPSS (Figure 2c) indicates that the amplitudes of the inhomogeneous absorption for the two formation mechanisms are similar for the given process parameters.

**Figure 3:**
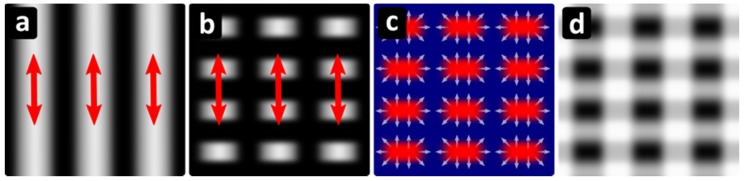
Schematic of the simultaneous formation of LIPSS and DLIP structures. (**a**) DLIP pattern on the surface. White represents intensity maxima and the red arrows the direction of the polarization. (**b**) Absorbed light intensity on the surface taking into account surface plasmon polariton excitation. (**c**) Hot (red) and cold (blue) areas resulting from absorption of light intensity pattern shown in (b). White arrows indicate the expected direction of the material flow. (**d**) Surface relief after resulting from the movement in (c). White indicates pronounced parts of the surface.

The schematic of the proposed formation process of the 2D structures is presented in Figure 3. Figure 3a shows the interference modulation of the intensity, corresponding to Λ_DLIP_ = 1300 nm. The white areas indicate the illuminated areas and the red arrow represents the direction of the laser’s polarization. Figure 3b shows the absorbed intensity on the surface assuming surface plasmon polariton excitation only along the illuminated areas. The period of the inhomogeneous light absorption was chosen accordingly to our experimental data (Λ_SPP_ = 900 nm). In Figure 3c, a schematic of the temperature of the surface after irradiation is presented. Areas that have absorbed light are hot (red) and areas which are not illuminated are cold (blue). White arrows indicate the directional flow from hot to cold areas as described in [25]. Lastly in Figure 3d, a grayscale topographic representation of the surface is shown considering the material displacement indicated by the white arrows in Figure 3c, where the white areas represent the higher surface areas.

### 3.2. Polyimide

**Figure 4:**
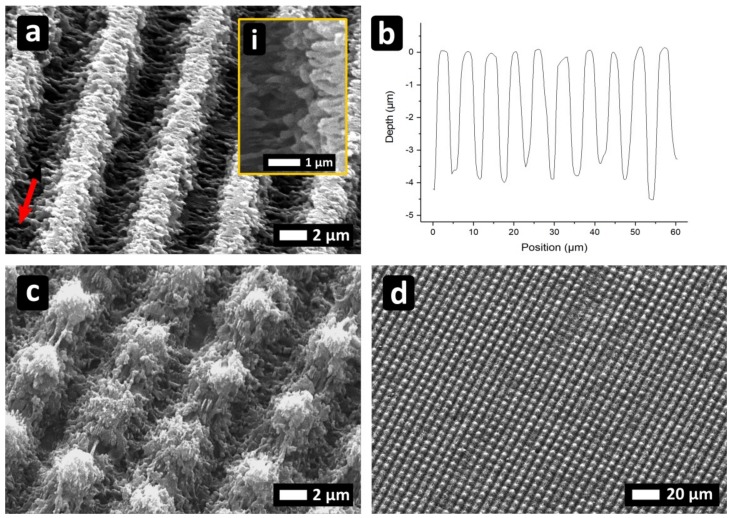
(**a**,**b**) Line-like periodic patterns on PI produced with N =10 and Φ = 1.34 J/cm². (**c**,**d**) Cross-like geometry obtained with N = 10 and N = 5 for the second irradiation. The arrow in (a) indicates the beam polarization direction.

PI foils were processed with Λ_DLIP_ = 6303 ± 16 nm, Φ = 0.4 J/cm^2^ and I_⊥_P. As it can be seen in Figure 4a, very well-defined, line-like structures could be obtained for N = 10. A two-photon absorption process can be considered as the main interaction mechanism [27], since the material is transparent to the employed IR wavelength [27,28,29,30,31]. Additionally, the structure depth of the patterns was approximately 3.9 µm (Figure 4b), which is much deeper compared to patterns with the same period obtained with ns pulses and UV (355 nm) irradiation, for which the material is a good absorber. Figure 4a also shows some additional periodic features, especially at the interference maxima positions. These features are perpendicular to the polarization direction, with Λ_LSFL_ = 856 ± 12 nm, which can be identified as LSFL (Figure 4a, inset “i”). On top of the minima positions, also some re-deposition of the ablated material can be observed.

Moreover, by rotating the PI-foils by 90°, cross-like patterns were also produced (Figure 4c). Furthermore, by translating the sample in X and Y direction using the procedure described in Reference [22], large areas can be also processed with high homogeneity (Figure 4d).

### 3.3. Sapphire

**Figure 5:**
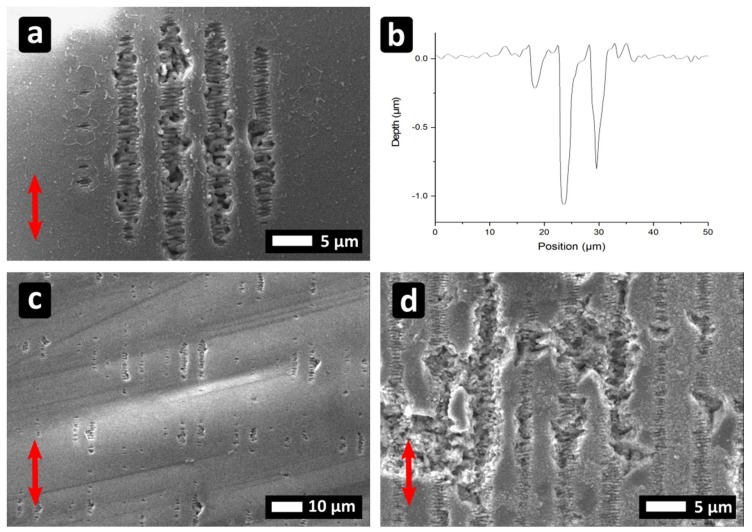
Line-like periodic patterns on sapphire produced with (**a**,**b**) N = 10 at a fluence of 1.80 J/cm²; (**c**) N = 5 with a pixel displacement of 25 µm and (**d**) N = 5 pulses with a pixel displacement of 10 µm at a fluence of 1.96 J/cm². The arrows indicate the beam polarization direction.

Finally, sapphire substrates were treated with Λ_DLIP_ = 5.7 µm and I_⊥_P. In Figure 5a, N = 10 and Φ = 1.80 J/cm² were employed. In the interference maxima, where ablation occurs, LSFL were obtained oriented perpendicular to laser polarization with Λ_LSFL_ = 520 ± 9 nm. At the central position of the treated area within the interference maxima, a depth of ~1.1 µm could be achieved (Figure 5b). According to previous investigations based on fused silica [32,33,34], also in this case the material is transparent at the laser wavelength and thus the absorption mechanism can be explained by a multi-photon absorption process. As already encountered on fused silica [35,36], and as recently reported also on sapphire [37], LIPSS can be produced on transparent materials when ultrashort pulses are employed, but the parameter window in which the structuring takes place may be limited.

In Figure 5c,d, the surface was processed with spots of N = 5 and Φ = 1.96 J/cm². The lateral and vertical displacement was 25 µm and 10 µm, respectively. Surface structures resulting from an extended DLIP processing can be observed only at the material positions presenting defects, which is typical for this material. On the other hand, for higher doses, the substrates can be significantly damaged due to Coulomb explosion [38] (see Figure 5d).

As demonstrated by Sedao et al. [39], the orientation of the HSFL can be dependent on the crystallographic orientation of the material, as shown for instance on nickel substrates employing low laser fluences. For all experiments regarding the structuring of Sapphire, the polarization of the laser beams was chosen to be parallel to the interference lines in order to produce ripples perpendicular to the orientation of the interference pattern. It is important to highlight that none of the produced patterns appeared to be affected by the crystallographic orientation of the Sapphire. In fact, the geometry and the period of the DLIP pattern is imposed by the interference modulation, while the LSFL are forming on Sapphire with the same characteristics as in PI, where no ordered crystallographic structure is present.

## 4. Conclusions

Several conclusions can be drawn, when DLIP and LIPSS surface patterning is applied simultaneously. Firstly, on stainless steel samples, when Λ_DLIP_~Λ_LSFL_, higher fluence levels to favor the DLIP generation whereas at low fluencies predominantly LIPSS are generated. When both mechanisms act in the same direction (I_∥_P), the DLIP and LSFL interactions are in competition and the process parameters define the predominant structures. In the perpendicular configuration (I_⊥_P), the two interaction mechanisms are superimposed producing a pillar-like morphology in a novel single-step process. This configuration generates 2D topographies, where the periodicities in the two directions can be controlled independently. Noticeable changes in DLIP and LSFL periods are attributed to the interplay between the two interaction mechanisms and should be further investigated. For sapphire and polyimide, the structuring mechanism can be explained by a multi-photon absorption process, since both materials are transparent at the laser wavelength. The DLIP-structures present both a considerable depth and LSFL can be also found with a material-dependent periodicity (856 nm and 520 nm, for PI and sapphire respectively).

It is important to state that the combination of the sub-picosecond laser radiation with the interference patterning created unique microstructures, which cannot be replicated otherwise. Further works will be carried out in order to assess the wettability changes on polymers, employing the high-aspect ratio and highly rough microstructures which has been produced on polyimide. On the other hand, tribological analyses can be carried out on steel surfaces textured with pillar-like structures in the micro- or sub-micrometer range, as produced in this work. Moreover, the hierarchical microstructures created in this work on transparent materials can open new horizons in the field of the fabrication of highly sophisticated optical security elements.

## Figures and Tables

**Table 1:** Range of the experimental values used.

Value	Stainless-Steel	Polyimide	Sapphire
Fluence Φ [J]	0.06–0.28	1.34	1.80–1.96
Interference Area [µm]	~100, ~25	~100	~100
λDLIP [μm]	6.3, 1.4	6.3	5.7
N	20–50	5–10	5–10
Pulse distance [μm]	5–100	5–20	10–100
